# Predictability of Deep Bite Correction and Curve of Spee Flattening in Clear Aligner Therapy: An Open-Label and One Arm Retrospective Study

**DOI:** 10.3390/healthcare14040548

**Published:** 2026-02-23

**Authors:** Alessandro Nota, Floriana Bosco, Laura Pittari, Chiara Clerici, Miryam Romito, Francesco Manfredi Monticciolo, Giorgio Gastaldi, Simona Tecco

**Affiliations:** Dental School, Vita-Salute San Raffaele University and IRCCS San Raffaele Hospital, 20132 Milan, Italy; boscofloriana@gmail.com (F.B.); laura_pittari@hotmail.it (L.P.); chia.clerici@gmail.com (C.C.); m.romito@studenti.unisr.it (M.R.); francesco.manfredi.monticciolo@gmail.com (F.M.M.); gastaldi.giorgio@hsr.it (G.G.); tecco.simona@hsr.it (S.T.)

**Keywords:** clear aligners, deep bite, Spee curve, treatment planning, predictability, digital dentistry

## Abstract

Objectives: This study aims to evaluate the predictability of Clear Aligner Therapy (CAT) in deep bite correction and Spee Curve flattening by comparing final intraoral scans with planned outcomes in ClinCheck. Methods: STL files from pre-treatment, post-treatment (first aligner cycle), and planned final positions of 18 patients (12 females; 6 males; mean age 30.9 ± 12.3 years) were analyzed. The software Medit Link (version 3.4.4) was used to measure overbite as the vertical distance between the incisal edges of the maxillary and mandibular central incisors and the Curve of Spee in both arches by drawing a reference line between the most distal molar and the central incisor on each side, recording the perpendicular distance from the distal cusp. Measurements were repeated on post-treatment and ClinCheck STL files. Data analysis was performed using a Student’s *t*-test (*p* = 0.05) to compare the expected and actual measure variations and intraclass correlation coefficient (ICC) to assess aligner predictability. Results: A significant discrepancy was observed in overbite correction (55% achieved), with a significant difference between expected and actual outcomes (*p* = 0.0001). Moderate differences were noted for the lower Spee Curve (62% achieved), while the upper Spee Curve showed 86% of the expected change. ICC values were moderate for overbite and lower Spee Curve, and good for the upper Spee Curve. Conclusions: ClinCheck overestimates deep bite correction. Upper Curve of Spee flattening is highly predictable, while the lower curve flattening has lower predictability.

## 1. Introduction

In recent years, the increasing demand for orthodontic treatment among adult patients has driven the development of alternative solutions that provide enhanced esthetics and comfort compared to conventional fixed orthodontic therapies [1]. Clear Aligner Therapy (CAT) is increasingly widespread among orthodontists as a preferred treatment option [2,3], offering advantages such as reduced chair time, fewer visits, easier oral hygiene maintenance, improved esthetics, and lower risk of periodontal disease [4,5].

The most widely used and prescribed CAT system is Invisalign^®^ (Align Technology, San Jose, CA, USA), which was introduced in 1998 [3,4,6,7]. This development is part of a broader ongoing transformation in orthodontics, characterized by the integration of transparent thermoplastic materials, digital innovations such as Computer-Aided Design and Computer-Aided Manufacturing (CAD/CAM), stereolithography, and software tools for simulating tooth movements, including ClinCheck^®^ Pro (Align Technology, Inc.) [7].

Through the digital planning software, aligner producers use digital treatment planning, allowing for the simulation of desired tooth movements and the establishment of an ideal final tooth position [3,4,8]. This software provides graphical representations and numerical data, including overjet, overbite (OVB), and the Curve of Spee, facilitating the monitoring and optimization of the patient’s occlusal characteristics throughout treatment [3,4,8].

The software also supplies specific strategies for managing deep bite and open bite, a prevalent occlusal issue in adult patients [9,10].

Deep bite, characterized by an excessive overlap of the maxillary and mandibular teeth, presents one of the most challenging cases to manage using clear aligners [7].

Treatment typically involves the intrusion of incisors, the extrusion of molars, or both, depending on the degree of incisal show [7]. In deep bite cases, it is possible to plan anterior incisor intrusion and/or posterior tooth extrusion, potentially accompanied by proclination of the upper and lower incisors to reduce excessive overbite and flatten the Curve of Spee [9,10]. Additionally, posterior attachments help stabilize the aligner during intrusion, while precision bite ramps can aid in achieving proper occlusion in more complex situations, with overcorrections applied to enhance predictability [7,9,11,12].

However, it is essential to recognize that what is graphically depicted in the software may not always be clinically achievable [8,13]. Numerous investigations have indicated that the initial treatment plan often requires one or more refinements, incorporating additional aligners to meet the originally prescribed goals [4,14,15,16,17,18]. Consequently, special attention must be given to virtual planning aspects, such as the placement and shape of attachments, movement staging, knowledge of aligner biomechanics, and the potential need for overengineering—emphasizing certain movements in the virtual plan [13].

The aim of this study is to analyze the predictability of treatment with clear aligners in correcting deep bite by comparing the final intraoral scans with the planned final positions in ClinCheck. Additionally, the study will examine both predicted and actual variations in deep bite and Curve of Spee, providing insights into the effectiveness of CAT in these specific cases. The null hypothesis of this study was that no significant differences would be observed between the planned outcomes programmed in ClinCheck and the actual clinical outcomes achieved at the end of the first aligners set, in terms of overbite correction and Curve of Spee flattening.

## 2. Materials and Methods

A single-center open-label retrospective study without a control group was conducted on a sample of patients with deep bite, treated with CAT. This study was conducted by using the STROBE Statement Checklist. The study was approved by the Ethic Committee of the San Raffaele University (38/INT//2023 of 12 July 2023). The study was conducted at Vita-Salute San Raffaele University in Milan, Italy. For each patient, pre-treatment STL (Standard Tessellation Language), final STL following the completion of the first aligner cycle, and the final position programmed in the first clinician-approved Clincheck (accessed at https://clincheck.invisalign.com/ in 2024) plan (in STL) were used. Subsequent stages involving additional aligners were not considered.

### 2.1. Sample

A total of 18 patients (12 females; 6 males; mean age 30.9 ± 12.3 years) treated with Invisalign^®^ in a private dental practice were included in this sample.

Patients were selected according to the following inclusion criteria: full permanent dentition; patients undergoing orthodontic treatment with Invisalign^®^ (Align Technology, San Jose, CA, USA), on both arches; availability of pre-treatment and post-treatment digital scans; pre-treatment overbite between 3 and 8 mm; non-extraction orthodontic treatment. While the exclusion criteria considered were: patients requiring orthognathic surgery; patients with an overbite greater than 8 mm; patients with any type of dental movement disorder; patients with craniofacial malformations.

Patients were consecutively selected. Follow-up corresponded to the completion of the first aligners set.

**Figure 1:**
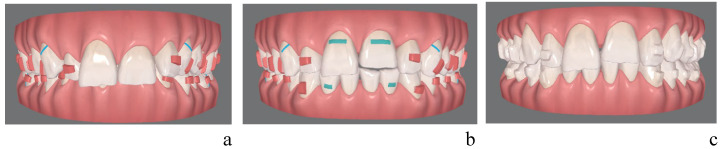
(**a**) Initial ClinCheck, first set of aligners. (**b**) Final ClinCheck, first set of aligners. (**c**) Initial ClinCheck, second set of aligners.

Figure 1 shows the initial (Figure 1a) and final (Figure 1b) ClinCheck of the first set of aligners, as well as the initial ClinCheck of the second set (Figure 1c) for a patient with deep bite. The Curve of Spee flattening was planned combining molar extrusion and incisor intrusion. A precise proportion between these two components was not planned for any case as each case requires a different esthetical and biomechanical approach that could lead to planning a more posterior intrusion or anterior intrusion.

### 2.2. Variables

The primary outcomes of this study were overbite (OVB) and the Curve of Spee in both the upper and lower arches. Overbite was defined as the vertical distance between the incisal edges of the maxillary and mandibular central incisors, with positive values indicating a deep bite. The Curve of Spee was defined as the perpendicular distance from the cusp of the most distal tooth to a line connecting the central incisor and the most distal molar in each arch. No other predictors, confounders, or effect modifiers were considered in this study.

### 2.3. Procedures

For the present study, pre- and post-treatment STL, as well as the expected outcome from ClinCheck, were imported into Compare©, an add-in integrated within the Medit Link software (version 3.4.4) Medit spa, Seoul, Republic of Korea), to analyze the models measuring the considered variables (Figure 1). Pre-treatment and post-treatment scans were acquired using the iTero scanner (Align Technology, San José, CA, USA), which utilizes structured light technology to provide high accuracy and reproducible digital models. All scans were performed following a standardized scanning protocol by experienced operators. Any scanning errors were considered clinically negligible.

Patients wore each aligner for one week, following the standard Invisalign^®^ protocol. The duration of the first aligner set lasted on average 9 months, and only this cycle was considered in the study.

**Figure 2:**
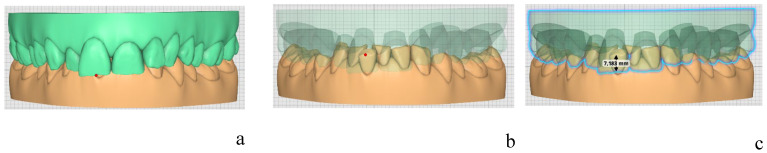
(**a**) Landmark on the edge of the upper central incisor. (**b**) Landmark on the edge of the lower central incisor. (**c**) Measurement of overbite by connecting these two points.

Firstly, pre-treatment STL was imported into Compare©, where overbite was measured as the vertical distance between the incisal edges of the maxillary central incisors and the incisal edges of the mandibular central incisors, with a positive value indicating a deep bite (Figure 2a–c). Figure 2a illustrates the landmark of the upper central incisor, while Figure 2b shows the landmark of the lower central incisor. These two points were connected to measure the overbite, as shown in Figure 2c.

Patients with Class II malocclusion and a deep bite between 3 and 8 mm were included in the study. Next, the Curve of Spee was measured in both the upper and lower arches, by drawing a line between the last molar and the central incisor for each side and for each arch, recording the perpendicular distance from the cusp of the most distal tooth to this line.

**Figure 3:**
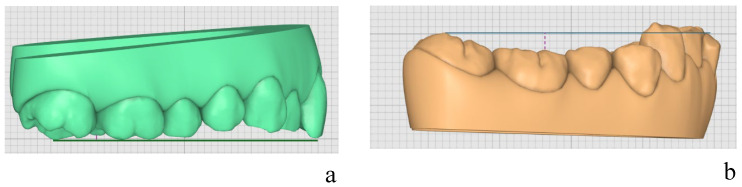
(**a**) Measurement of the upper Curve of Spee: the green line represents the reference line between the last molar and the central incisor, while the red line indicates the perpendicular distance from the cusp of the most distal tooth to the reference line. (**b**) Measurement of the lower Curve of Spee: the blue line represents the reference line between the last molar and the central incisor, while the red dashed line indicates the perpendicular distance from the cusp of the most distal tooth to the reference line.

The measurement of the upper Curve of Spee is shown in Figure 3a, while Figure 3b illustrates the measurement of the lower Curve of Spee.

The same measurements were repeated on the post-treatment STL (Figure 1b), and on the final position programmed in the first clinician-approved Clincheck (STL) (Figure 1c).

**Figure 4:**
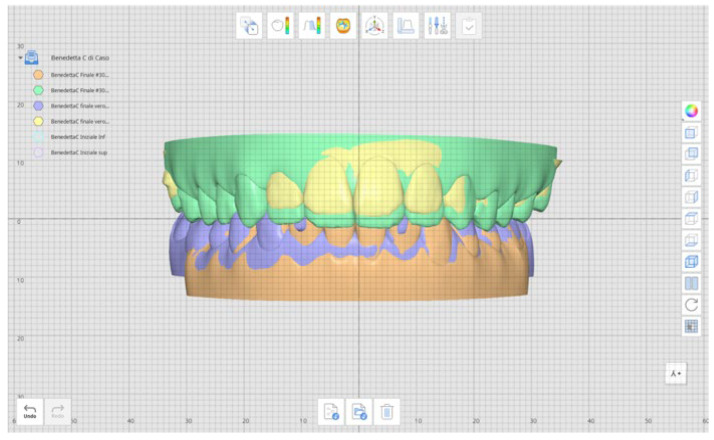
Comparison between the actual result (purple and yellow model) and the expected result (green and orange model).

Figure 4 shows the superimposition of the post-treatment STL with the final position programmed in the first clinician-approved Clincheck STL.

Each parameter was measured independently by three operators. Each operator performed the measurements twice, and the average values were used to assess reliability.

Patients were selected based on predefined inclusion and exclusion criteria, specifically considering the presence of deep bite. While all participants met the study requirements, it may introduce selection bias as the sample was not randomly chosen. To minimize measurement and observer bias, all STL measurements were performed independently by two operators, and each measurement was repeated twice to assess reliability. The intraclass correlation coefficient (ICC) was calculated to evaluate the consistency of the measurements showing an excellent reliability (ICC = 0.95). The two operators already had experience in managing and measuring these variables on STL models and were supervised by a third operator with more than 15 years of orthodontic experience.

Following the measurements performed on Medit link software, a descriptive analysis of the data was conducted using Microsoft Excel (Microsoft Office). After verifying the normality of the data distribution, a Student’s *t*-test was applied to assess the differences between the considered variables, with a significance threshold set at *p* = 0.05. Additionally, the intraclass correlation coefficient (ICC) was calculated on the variations in the parameters to evaluate the predictability of the outcome. No subgroup analyses, sensitivity analyses, or missing data were present.

The sample size was calculated with a power analysis performed on the first 8 cases included in the study, aiming to achieve a power of 80% and an alpha error of 5% on the primary outcome. The analysis showed that a minimum of 10 subjects were necessary in order to achieve the statistical power.

## 3. Results

A total of 18 patients were initially assessed, and all met the inclusion criteria; therefore, no patients were excluded. All 18 patients were included in the analysis. As previously mentioned, the study sample included 12 females and 6 males, with a mean age of 30.9 ± 12.3 years. All patients presented with Class II malocclusion and deep bite between 3 and 8 mm. No missing data was present. Follow-up corresponded to the completion of the first aligner cycle for all patients.

Table 1 presents the mean values and standard deviations (SDs) of OVB and the upper (SpeeU) and lower (SpeeL) Spee Curves for the sample population at pre-treatment (T0), post-treatment (T1R), and the expected outcome (T1C). Before treatment, the patients had an average overbite of 6.11 ± 1.16 mm, lower Spee Curve of 2.21 ± 1.20 mm, and upper Spee Curve of 0.49 ± 0.50 mm.

At T1R, the mean values were 3.94 ± 1.10 mm for overbite, 1.36 ± 0.68 mm for lower Spee Curve, and 0.11 ± 0.23 mm for upper Spee Curve. The predicted values at T1C were 2.19 ± 0.81 mm, 0.92 ± 0.56 mm, and 0.04 ± 0.13 mm for overbite, lower Spee Curve and upper Spee Curve, respectively.

Statistical analysis revealed significant differences in the overbite and lower Spee Curve parameters, while no significant differences were observed for the upper Spee Curve parameter.

Table 2 presents the mean changes in the measured parameters from T0 (pre-treatment) to T1 (end of treatment), along with the predicted change in ClinCheck.

For overbite, the expected change was 3.92 ± 1.20 mm, while the actual change achieved was 2.17 ± 1.16 mm. The expected mean change for the lower Spee Curve was 1.38 ± 0.97 mm, whereas the actual change was 0.85 ± 1.02 mm. Similarly, for the upper Spee Curve, the expected correction was 0.44 ± 0.44 mm, while the actual change was 0.38 ± 0.42 mm.

Statistical analysis revealed no significant differences between expected and actual changes for the upper and lower Spee Curves. However, a significant difference was observed for the overbite (*p* = 0.0001), indicating a discrepancy between the predicted and achieved outcomes.

These results are supported by the ICC values calculated for the parameter changes, which were classified as moderate for overbite and good for lower and upper Spee Curves. The ICC classification thresholds were as follows: poor (<0.50), moderate (0.50–0.75), good (0.75–0.90), and excellent (>0.90).

Overall, the percentage difference between the actual and the expected changes was 55% for overbite, 62% for the lower Spee Curve, and 86% for the upper Spee Curve. In other words:

- On average, 55% of the expected overbite correction was achieved.
- For the lower Spee Curve, 62% of the expected leveling was accomplished.
- For the upper Spee Curve, an average of 86% of the expected leveling was reached.

**Figure 5:**
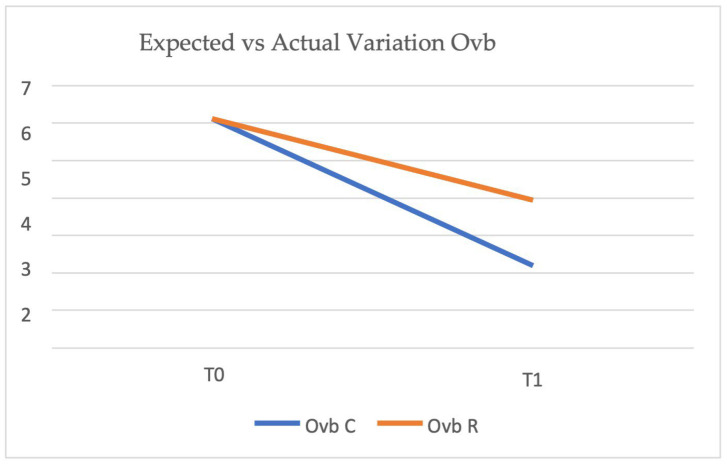
Expected (Ovb C, blue line) vs. actual (Ovb R, orange line) overbite variation from T0 (pre-treatment) to T1 (post-treatment). The graph illustrates the difference between the predicted reduction in overbite, as programmed in ClinCheck, and the actual outcome achieved.

Figure 5 shows a comparison between expected and actual overbite variation from T0 (pre-treatment) to T1 (post-treatment). The blue line (Ovb C) represents the predicted overbite reduction according to ClinCheck, while the orange line (Ovb R) represents the actual overbite reduction achieved. The expected overbite was planned to decrease from 6.11 mm to 2.19 mm, whereas the actual reduction reached 3.94 mm, indicating a discrepancy between the predicted and achieved outcomes.

Table 3 represents the mean overbite values at T0 and T1 for both the expected (Ovb C) and actual (Ovb R) variations.

**Figure 6:**
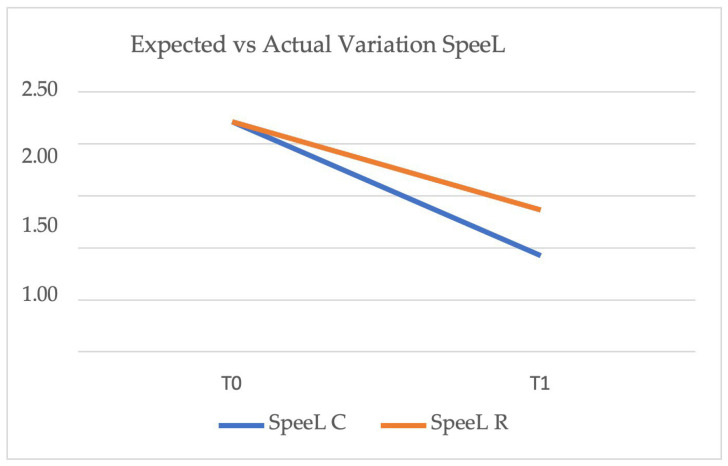
Expected (SpeeL C, blue line) vs. actual (SpeeL R, orange line) lower Spee Curve variation from T0 (pre-treatment) to T1 (post-treatment). The graph illustrates the difference between the predicted reduction, as programmed in ClinCheck, and the actual outcome achieved.

Figure 6 shows a comparison between the expected and actual lower Spee Curve variation from T0 (pre-treatment) to T1 (post-treatment). The blue line (SpeeL C) represents the predicted lower Spee Curve according to ClinCheck, while the orange line (SpeeL R) represents the actual outcome achieved. The expected lower Spee Curve was planned to decrease from 2.22 mm to 0.92 mm, whereas the actual reduction reached 1.36 mm, indicating a minor discrepancy between the predicted and achieved outcomes.

Table 4 represents the mean lower Spee Curve values at T0 and T1 for both the expected (SpeeL C) and actual (SpeeL R) variations.

**Figure 7:**
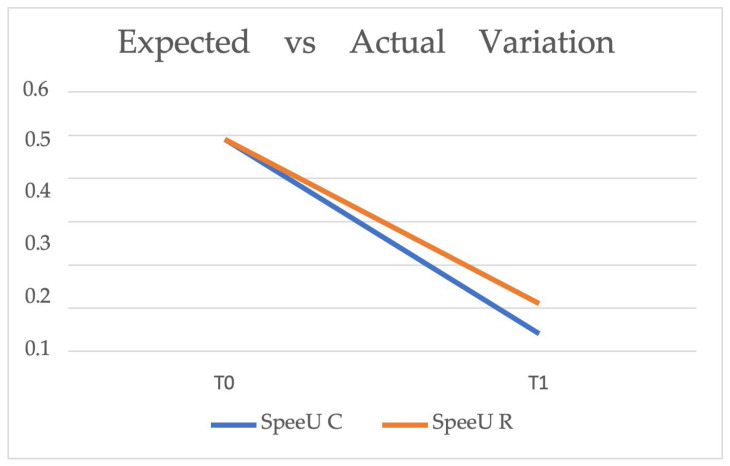
Expected (SpeeU C, blue line) vs. actual (SpeeU R, orange line) upper Spee Curve variation from T0 (pre-treatment) to T1 (post-treatment). The graph illustrates the difference between the predicted reduction, as programmed in ClinCheck, and the actual outcome achieved.

Figure 7 illustrates the comparison between the expected and actual upper Spee Curve variation from T0 (pre-treatment) to T1 (post-treatment). The blue line (SpeeU C) represents the predicted upper Spee Curve according to ClinCheck, while the orange line (SpeeU R) represents the actual outcome achieved. The expected reduction was from 0.49 mm to 0.04 mm, while the actual reduction reached 0.11 mm. Although a slight discrepancy is observed, the result achieved is relatively close to the predicted outcome.

Table 5 presents the mean upper Spee Curve values at T0 and T1 for both the expected (SpeeU C) and actual (SpeeU R) variations.

## 4. Discussion

In recent decades, the Invisalign^®^ system has become widely adopted, both due to the improved esthetics and greater comfort it offers to patients [19,20], particularly adult patients who are increasingly seeking orthodontic treatment, and due to the numerous innovations that have made it possible to use this type of treatment not only for simple corrections but also for more complex malocclusions.

Among the complex malocclusions that can be treated with clear aligner systems are vertical issues, such as deep bite.

The aim of this study was to analyze the predictability of deep bite correction and leveling of the Curve of Spee as planned in digital treatment simulations.

It was observed that, for the correction of the upper Curve of Spee in cases of deep bite, there were no significant differences, both in the T1R-T1C comparison and in the respective variations. This indicates that the flattening of the upper Spee Curve is quite efficient, with approximately 86% of the planned flattening from ClinCheck being achieved by the end of treatment. Additionally, this correction percentage appears highly predictable, as evidenced by a good ICC (0.87).

In contrast, for the flattening of the lower Curve of Spee, the treatment efficiency is lower; indeed, the observed data suggest, with good predictability (ICC = 0.86), that the treatment achieves an actual outcome that is, on average, only 62% of the planned result.

Similarly, for overbite, the observed data suggests moderate efficiency, as the treatment leads to an actual correction that is approximately 55% of what was planned. Furthermore, this percentage is moderately predictable, according to Koo, T. K.’s classification, with an ICC of 0.55 [21].

Based on the present findings, the null hypothesis was rejected for overbite correction and lower Curve of Spee flattening, while it was not rejected for the upper Curve of Spee, where no statistically significant differences between planned and achieved outcomes were observed.

Blundell et al. (2021) assessed the predictability of overbite correction with Invisalign^®^ and found that ClinCheck predicts a greater reduction in overbite than what is clinically achieved in 95.3% of patients [14].

In 2022, the same authors divided the sample of 42 patients into two treatment groups: one group receiving treatment with Invisalign^®^, including bite ramps and aligners made from the new SmartTrack material, and another group with aligners produced from the old EX30 material, without bite ramps. The Invisalign^®^ system achieved 43.4% of the overbite reduction planned in ClinCheck in the group with bite ramps, and 55.1% in the group treated with aligners made from EX30 without bite ramps [15].

Goh et al. assessed the predictability of Curve of Spee leveling in a 2022 study, which found that ClinCheck overestimates leveling in 86% of patients, and that the lower first molars exhibit the least amount of programmed extrusion compared to their original position [16].

Shahabuddin et al., in a 2022 study, evaluated the extent of overbite correction in a group of 24 patients treated with aligners, which was found to be an average of 33% [17].

Kravitz analyzed the effectiveness of dental movements with Invisalign^®^, and the results of his study indicated that the least effective movement was extrusion, while the average effectiveness of intrusion movements was 41.3% [18].

In a recent study published in 2024, Jessica K. et al. assessed the effectiveness of clear aligners for correcting deep bite by comparing the outcomes of the first set of aligners with those of subsequent sets. They observed a 1.25 mm improvement in overbite with the first set, significantly less than the initially planned 3.27 mm, resulting in a mean treatment accuracy of 37.6%. Furthermore, the study found that the efficacy of treatment decreased with the use of later aligner sets [22].

Therefore, it can be concluded that the data in the literature appear to be consistent with the results of the present study. Regarding the fact that the efficiency of leveling the upper Curve of Spee was greater than that of the lower Curve of Spee, it should be noted that the average planned flattening for the upper Spee Curve in the cases considered was considerably less than that required for the lower Spee Curve, being under one millimeter. This may have contributed to the greater efficiency observed for the upper Spee Curve (86%) compared to the lower Spee Curve (62%).

The present study has several limitations, including the small sample size, the limited depth of the upper Curve of Spee in the treated group, and no data on genetic or epigenetic factors.

Additionally, the effectiveness of certain variables in the clinical protocols should be analyzed, as they could enhance the efficacy and outcome of treatment with aligners as well as the comparison with other aligner producers. For example, the addition of bite ramps (thickenings incorporated on the palatal surface of the aligners at the level of the upper central, lateral, or canine incisors) that allow for an increased degree of flattening of the Curve of Spee and intrusion of the anterior segment, as well as of specific treatment staging. Measuring the variables of the present study, it was impossible to analyze the predictability in anterior intrusion or posterior intrusion components in Curve of Spee flattening and overbite correction.

It should be considered as a limitation also that the present study only deals with dental variables and does not consider the presence/absence of a specific vertical skeletal pattern.

In this study, all scans were performed using a standardized protocol and any scanning errors were considered clinically negligible; in fact, the aligners produced from the scans had a perfect fitting on dental arches. To minimize measurement and observer bias, all STL measurements were performed by experienced operators, ensuring that the measurements used for analysis were reliable.

In 2024, Meade et al. examined planned and achieved changes in overbite (OB) and overjet in adolescent patients treated with Invisalign^®^. The study found that less than 60% of the planned OB changes were achieved, with 58.33% of the planned increase in OB and 55.55% of the planned reduction in OB being accomplished. These results indicate significant discrepancies between the predicted and actual movements achieved with aligners [3,4].

It is likely that with the addition of further phases, it would be possible to get closer to the results predicted by ClinCheck. Align Technology has reported that 20–30% of patients treated with Invisalign^®^ may require adjustments to the treatment plan during therapy or the addition of aligners for case refinement to achieve the originally set objectives. These results are consistent with what was reported in a recently published systematic review about deep bite correction with CAT [23].

Consistent with the existing literature, the data obtained from the present study confirm that in cases of deep bite, the extent of correction achieved by the end of treatment is lower than that planned in ClinCheck, reaching only 55%. One possible explanation is that posterior extrusion movements, which are crucial for correcting deep bite, are among the most challenging to achieve with aligners, according to the literature. Therefore, in deep bite cases, it may be beneficial to adopt an overengineering approach when adjusting ClinCheck settings to enhance posterior extrusion, aiming for more effective correction. Further studies with larger sample sizes and study designs are necessary to evaluate the efficacy of different staging protocols on the predictability of the planned results.

## 5. Conclusions

Within the limits of this study, it can be concluded that the Invisalign^®^ appliance seems to achieve 55% of the planned reduction in overbite compared to the outcome prescribed in the first ClinCheck plan. The efficacy in flattening the Spee Curve after the first ClinCheck plan is 62% for the lower Spee Curve while the flattening of the upper Spee Curve is highly predictable and has high efficiency. Therefore, the movements programmed in the ClinCheck software may not be completely clinically predictable in addressing the correction of deep bite and an overengineering of the ClinCheck plan could be advanced to obtain the target result.

## Figures and Tables

**Table 1:** Mean values of the measured variables and statistical analysis using a *t*-test to compare the value from the final scans (T1R) with those programmed in CliCheck (T1C).

		T0	T1R	T1C	*t*-Test (T1R vs. T1C)
**Ovb**	Mean Value	6.11	3.94	2.19	<0.0001
	SD	1.16	1.10	0.81	
**SpeeL**	Mean Value	2.21	1.36	0.92	0.038
	SD	1.20	0.68	0.56	
**SpeeU**	Mean Value	0.49	0.11	0.04	0.200
	SD	0.50	0.23	0.13	

**Table 2:** Comparison of expected variations (T0-T1C) and actual variations (T0-T1R) using a *t*-test, with ICC analysis to assess predictability.

		Expected Change (T0-T1C)	Actual Change (T0-T1R)	*t*-Test	ICC
**Ovb**	Mean Value	3.92	2.17	<0.0001	0.55
	SD	1.20	1.16		
**SpeeL**	Mean Value	1.38	0.85	0.120	0.86
	SD	0.97	1.02		
**SpeeU**	Mean Value	0.44	0.38	0.633	0.87
	SD	0.44	0.42		

**Table 3:** Mean overbite values at T0 and T1 for expected (Ovb C) and actual (Ovb R) variations.

	T0	T1
Ovb C	6.11	2.19
Ovb R	6.11	3.94

**Table 4:** Mean lower Spee Curve values at T0 and T1 for expected (SpeeL C) and actual (SpeeL R) variations.

	T0	T1
SpeeL C	2.21	0.92
SpeeL R	2.21	1.36

**Table 5:** Mean upper Spee Curve values at T0 and T1 for expected (SpeeU C) and actual (SpeeU R) variations.

	T0	T1
SpeeU C	0.49	0.04
SpeeU R	0.49	0.11

## Data Availability

The data that support the findings of this study are available from Vita-Salute San Raffaele University but restrictions are applied to the availability of these data, which were used under license for the current study, and so they are not publicly available. Data are, however, available from the authors upon reasonable request.

## Abbreviations

The following abbreviations are used in this manuscript:

CAT	Clear Aligner Therapy
STL	Standard Tessellation Language
ICC	Intraclass Correlation Coefficient
SpeeU	Upper Curve of Spee
SpeeL	Lower Curve of Spee
CAD/CAM	Computer-Aided Design and Computer-Aided Manufacturing
OB	Overbite
SD	Standard Deviation
